# Wide-eyed glare scares raptors: From laboratory evidence to applied management

**DOI:** 10.1371/journal.pone.0204802

**Published:** 2018-10-11

**Authors:** Martine Hausberger, Anthony Boigné, Clémence Lesimple, Laurine Belin, Laurence Henry

**Affiliations:** 1 CNRS, Laboratoire d’Ethologie animale et humaine, UMR 6552, Université de Rennes, Université de Caen-Normandie, France; 2 Société d’Accélération du Transfert de Technologies (SATT) Ouest Valorisation, Rennes, France; 3 Université de Rennes, Laboratoire d’Ethologie animale et humaine, UMR 6552, CNRS, Université de Caen-Normandie, France; Universita degli Studi di Pisa, ITALY

## Abstract

Raptors are one of the most important causes of fatalities due to their collisions with aircrafts as well as being the main victims of collisions with constructions. They are difficult to deter because they are not influenced by other airspace users or ground predators. Because vision is the primary sensory mode of many diurnal raptors, we evaluated the reactions of captive raptors to a “superstimulus” (a “paradoxical effect whereby animals show greater responsiveness to an exaggerated stimulus than to the natural stimulus”) that combined an “eye shape” stimulus (as many species have an aversion for this type of stimulus) and a looming movement (LE). This looming stimulus mimics an impending collision and induces avoidance in a wide range of species. In captivity, raptors showed a clear aversion for this LE stimulus. We then tested it in a real life setting: at an airport where raptors are abundant. This study is the first to show the efficiency of a visual non-invasive repellent system developed on the basis of both captive and field studies. This system deterred birds of prey and corvids through aversion, and did not induce habituation. These findings suggest applications for human security as well as bird conservation, and further research on avian visual perception and sensitivity to signals.

## Introduction

Diurnal raptors have the highest visual acuity of all bird species [1], in particular falcons and eagles that have an acuity of 160 and 140 c/deg respectively (i.e. a flying falcon can detect a 2cm object from 18m above) [2, 3]. Despite these visual abilities, birds fail to detect some substrates (e.g. glass windows) or detect approaching objects (e.g. aircraft) too late. Thus, they can collide with power lines, buildings, communication towers or wind plants (1850 raptors were electrocuted in the USA between 1986 and 1996 and 124 raptors are killed every year in a 252-turbine wind farm in Spain [4]. Between 1912 (first report) and 2002, reports of bird strikes mention 55 fatal accidents resulting in 276 deaths and 108 aircraft destroyed in the USA alone [5, 6] while 94743 collisions between aircraft and birds occurred between 1990 and 2007 in UK, Canada, and USA [7]. In France, 800 collisions between birds and civil aircrafts are reported annually with 15% of them are classified as serious incidents [8]. Birds of prey have been responsible for 47% and 100% of the fatalities with aeroplanes and with helicopters respectively, probably because of their large size [5, 6] (see also the « Hudson river » accident in 2009 due to Canada geese *Brenta canadensis*). About 1.2 billion USD is spent annually worldwide by air companies because of bird strikes [9].

While humans have long used devices, such as scarecrows, to repel birds from food resources, finding efficient bird deterrents has become an urgent issue since developing human use of the airspace and increasingly high constructions has enhanced human-bird conflict. Finding stimuli that reliably repel different bird species is a major scientific challenge with both security abs conservation issues.

Many devices have been developed to deter birds from agricultural land or airport areas, including acoustic, visual, chemical or even lethal systems. While some of them (acoustic stimuli) are efficient in the short-term, most are ineffective and birds rapidly become habituated to all of them within hours or days [10, 11]. Birds of prey show little or no fear of other airspace users and may even be attracted by the distress calls used in some acoustic systems [5, 12]. Hanging corpses or stuffed conspecifics proved useful in dispersing roosts of black vultures from communication towers, but raises ethical problems because the species is a protected one and the solution goes again conservation challenges [13].

The aim of the present study was to develop a way to repel birds (especially raptors) without harming them by merely inducing aversion for particular areas where their presence endangered both raptors’ and humans’ lives. Using systems that induce physical exclusion from sensitive areas raises few welfare problems [14].

Given the importance of raptors’ visual system we focused on a visual system. Several constraints had to be taken into account: 1) since birds cannot slow down while flying, the stimulus/object had to be detected from a distance (that varies in real life according to the respective speed of both the bird and the object [15]); 2) since most birds of prey have a narrow binocular visual field (20–35°) and their frontal vision is tuned for movement detection [16], a visual deterrent stimulus had to incorporate movement and be large enough to capture the birds’ attention [14]; 3) since raptors’ sensitivity to contrast is high, the stimulus had to be a highly contrasted [15, 17].

As mentioned by Philiben [18], research in this field requires a combination of experiments under controlled conditions (limiting the influence of external factors) and in the field. Therefore we first tested the reactions of captive raptors to a variety of visual stimuli including « eyespots » that deter birds from attacking lepidopterans (maybe as a result of contrast, [19, 20]), geometric shapes (spheres or stars) and looming movements. Looming stimuli were first described by Gibson (1958) [21] as a “uniform rate of approach accompanied by an accelerated rate of magnification” and are characterized by a rapid symmetrical expansion [22], these stimuli mimic impending collision [23]. Looming stimuli attract the attention of humans from a very early age [24, 25] and most species flee when an object approaches directly and quickly [26]. Neurophysiological studies of birds show the existence of « collision neurons » that respond to the time to collision [26–28]. The eyespot part was also designed on the basis of Stevens et al.’s (2008) [19] conspicuous signal hypothesis, maximizing contrast through a black circle within a white surrounding. Given the well-known difficulties to deter raptors, our objective was to identify a potential “visual superstimulus” that would attract their attention and repel them [10].

According to Brielert & Anderson (1985) [29], a superstimulus refers to a “paradoxical effect whereby animals show greater responsiveness to an exaggerated stimulus than to the natural stimulus”. For example, oystercatchers may brood gulls’ eggs instead of their own eggs that are half the size [30] and gulls are more attracted to eggs that are one and a half bigger than their real eggs [31]. We hypothesized here that the use of a stimulus with exaggerate pattern could also be perceived as deterrent by animals. Given the results obtained in captivity, we identified a “superstimulus” that appeared to potentialise the effects of eyespots and looming effects and evaluated its applicability under “real life” conditions of an airport where large populations of raptors were present and constituted the major source of collisions with aircraft. Although this is a case study, our results suggest that this visual stimulus could prove useful to elicit an aversion of raptors (but also other large birds like corvids) for at-risk areas.

## Methods

### Study 1: In captivity

**Fig 1:**
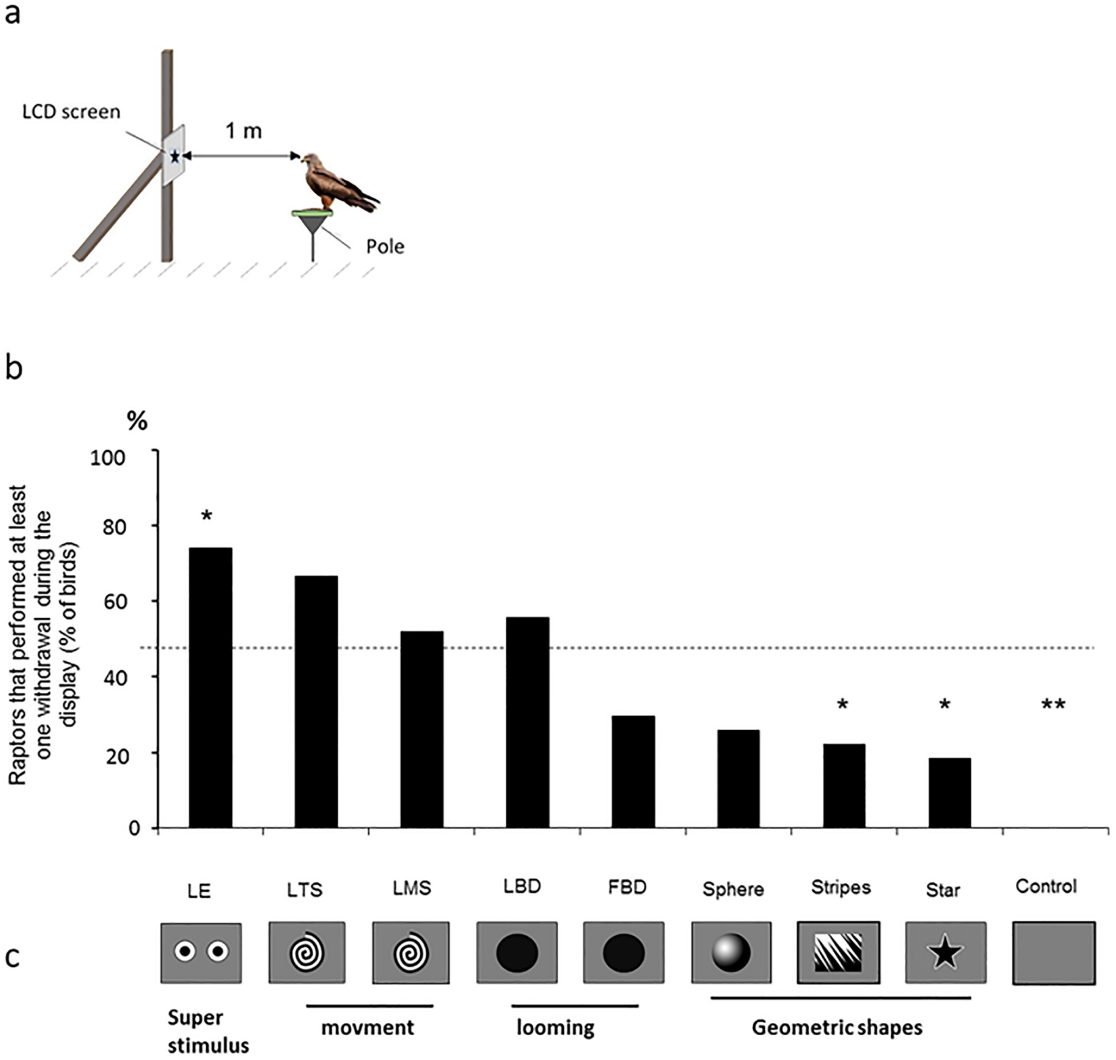
Captive study. a) test apparatus; b) birds (in percent) that reacted to the display of the visual stimulus. c) stimuli: LE: looming eyes, LTS: looming turning spiral (as used on some aircraft), LMS: looming moving spiral, LBD: looming black disc, FBD: static black disc, F Sph: static geometric shapes: sphere (depth impression), S: stripes (used for repelling birds from windows) [35], Fstar: static star, C control: grey screen. Chi-square test, * p < 0.05.; c) The stimulus displayed: LE: looming eyes, looming turning (LTS) versus moving (LMS) spiral, looming (LBD) versus static (FBD) black disc, static geometric shapes: sphere (F Sph), strips (S) static star (Fstar) and control (C).

This study took place at the Wildlife Jacana Studios (Sainte Fontaine, France) where captive animals are trained for movies. No specific permissions were required for this experiment (European directive 2010:/63/UE). Twenty-seven tame adult birds of prey belonging to 13 different species were presented 2D visual stimuli on a 15” LCD screen (Nec, AccuSync LCD52VM) while they were sitting on a pole 1 m away: 7 eagles (booted eagles *Hieraaetus pennatus*; golden eagles *Aquila chrysaetos*; tawny eagles *Aquila rapax*); 7 falcons (gyrfalcons *Falco rusticolus*, peregrine falcons *Falco peregrinus*, laggar falcons *Falco jugger*, saker falcons *Falco cherrug*), 5 black kites (*Milvus migrans*), 4 vultures (turkey vultures *Cathartes aura*, greater yellow-headed vultures *Cathartes melambrotus*), 2 bald eagles *Haliaeetus leucocephalus*, 2 hawks (Harris’s hawks *Parabuteo unicinctus*, black-chested buzzard-eagles (*Geranoaetus melanoleucus*). The birds were accustomed to being tethered on poles with a 1 m long leash (Fig 1a) and had resided in the facility for more than one year. Outside testing or training, they were released in large outdoor aviaries with water ad libitum and fresh meat every day.

Ten different visual stimuli were presented: looming eyespots (LE) and 8 with either a looming effect (looming black disc, looming and turning spiral, or looming and moving spiral) or static geometric shapes (a sphere or a star as well as stripes). Looming eyespots (LE) were supposed to be a potential “superstimulus” adding the effect of sudden exposure to eyespots, known to deter potential predators [32] and that of motion which, according to Tinbergen (1948) [30], when directed toward the animals, has an especially strong releasing value. Looming stimuli mimic imminent collision, and hence induce avoidance [33].

The control treatment was the screen without a stimulus (uniformly grey) (Fig 1c and S1 Fig). The looming stimulus was at 0.5 Hz with the stimulus completely filling the screen at the end. Preliminary studies determined that this speed elicits responses from birds. The stimuli were transmitted through Powerpoint from a laptop. Each stimulus was displayed once for 10s to each bird while the bird was facing the screen (S2 and S3 Figs). Each bird had one 1-hour session when the different stimuli were presented in a random order with intervals ranging from 25s to 10 min (every 2 to 5 occasions when the bird was facing the screen). The order was automatically generated so that the experimenter, who was standing 3m behind the screen, did not know which stimulus was being displayed. 270 tests (10 stimuli presented to 27 birds once) were performed and video recorded (Canon HG21) for analysis. Videos were analysed using continuous focal sampling a classical method in ethology [34] (record of all behavioural items occurring during the 10 seconds stimulus display and during the minute following display). Reactions (i.e. changes in behaviour) consisted in flight or turning away on the pole (see S2 Fig). The analyses were made twice by two observers who were blind to the stimuli presented (LB & CL) and they reached 100% agreement.

### Study 2: Field study

**Fig 2:**
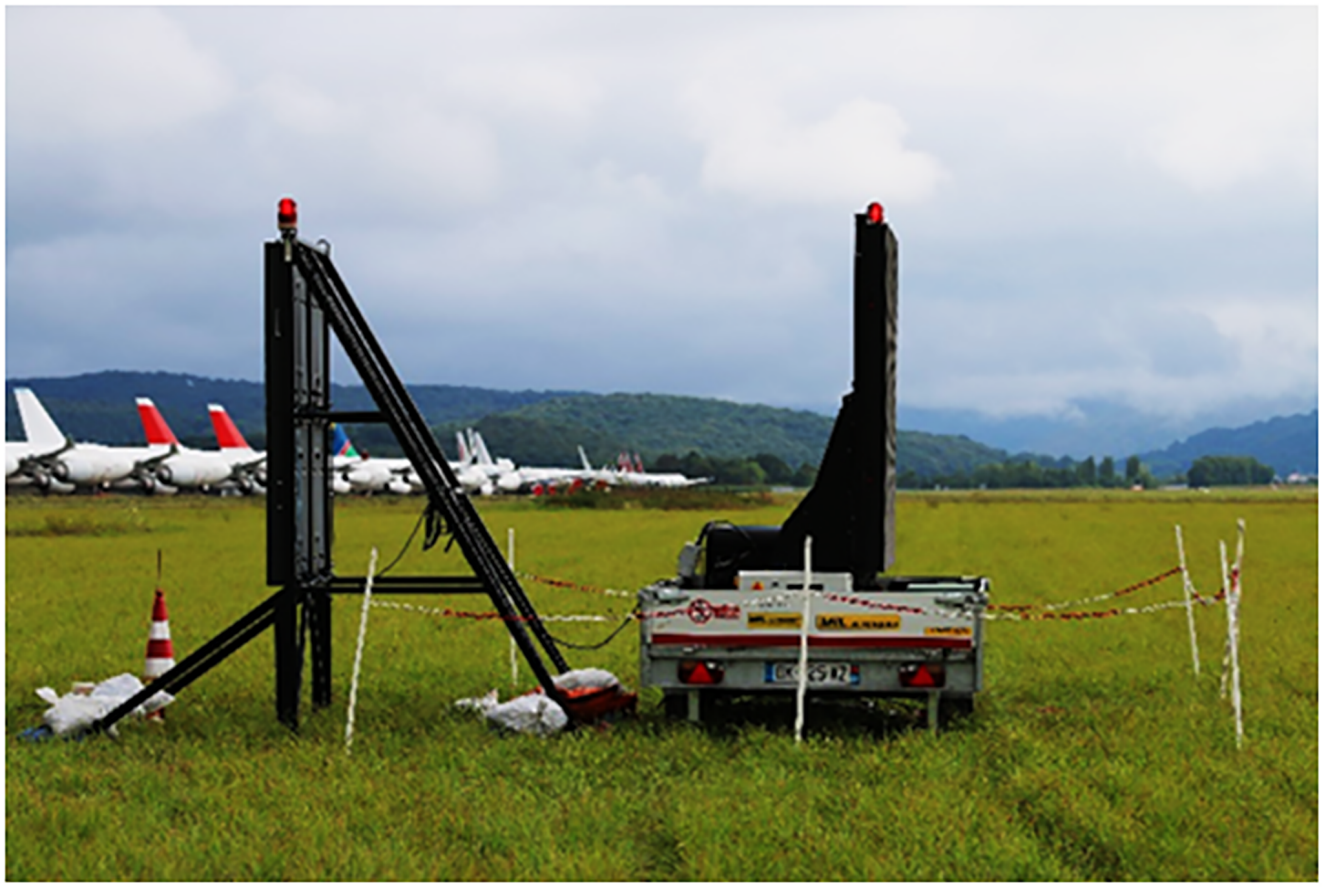
LED screens. The two screens placed back to back on the airport. The basis of both screens was positioned 1m above ground.

This study took place at Lourdes-Tarbes-Pyrénées airport in the South Western part of France between August 19th and September 29th 2016. No specific permissions were required for this experiment (European directive 2010:/63/UE). This field studies did not involve endangered or protected species. This airport attracts diurnal raptors and corvids because of the extensive agricultural activity both around and within the airport and has yearly records of bird strikes involving raptors at that time of year, even though most of them result in only moderate impacts. At this airport, as in all reports for other sites, most bird strikes occur around the runway [5]. The entire airport covers 120 ha, but has only one runway (3000m long and 45m wide) (able to receive B747 and A380 aircraft). The airport is surrounded by agricultural areas where hay and maize are produced. Large grass areas of the airport are exploited by local farmers. Between 2009 and 2013, 62% of the bird strikes reported were due to raptors (S4 Fig). Bird deterrents (sounds, gun shots, fireworks) are regularly used to scare birds especially during the 10 minutes before an aircraft lands or departs, but the local team reports no effect on raptors or corvids. Two 4x4m LED screens, especially designed for this purpose, were positioned so that the basis of the screen was 1m above ground (hence visible for a bird on a flat open ground) (Fig 2) at the most dangerous zone (landing and take-off area), one facing the runway, the other facing the field behind (to repel birds approaching the runway (Fig 2 and S5 Fig).

After two weeks habituating the local bird population to the presence of these screens with security lights, the program started (August 30th) and the looming eyespot stimulus was presented continuously from 6 am to 10 pm. In order to maximize contrast, the screen background was white in this second experiment.

**Fig 3:**
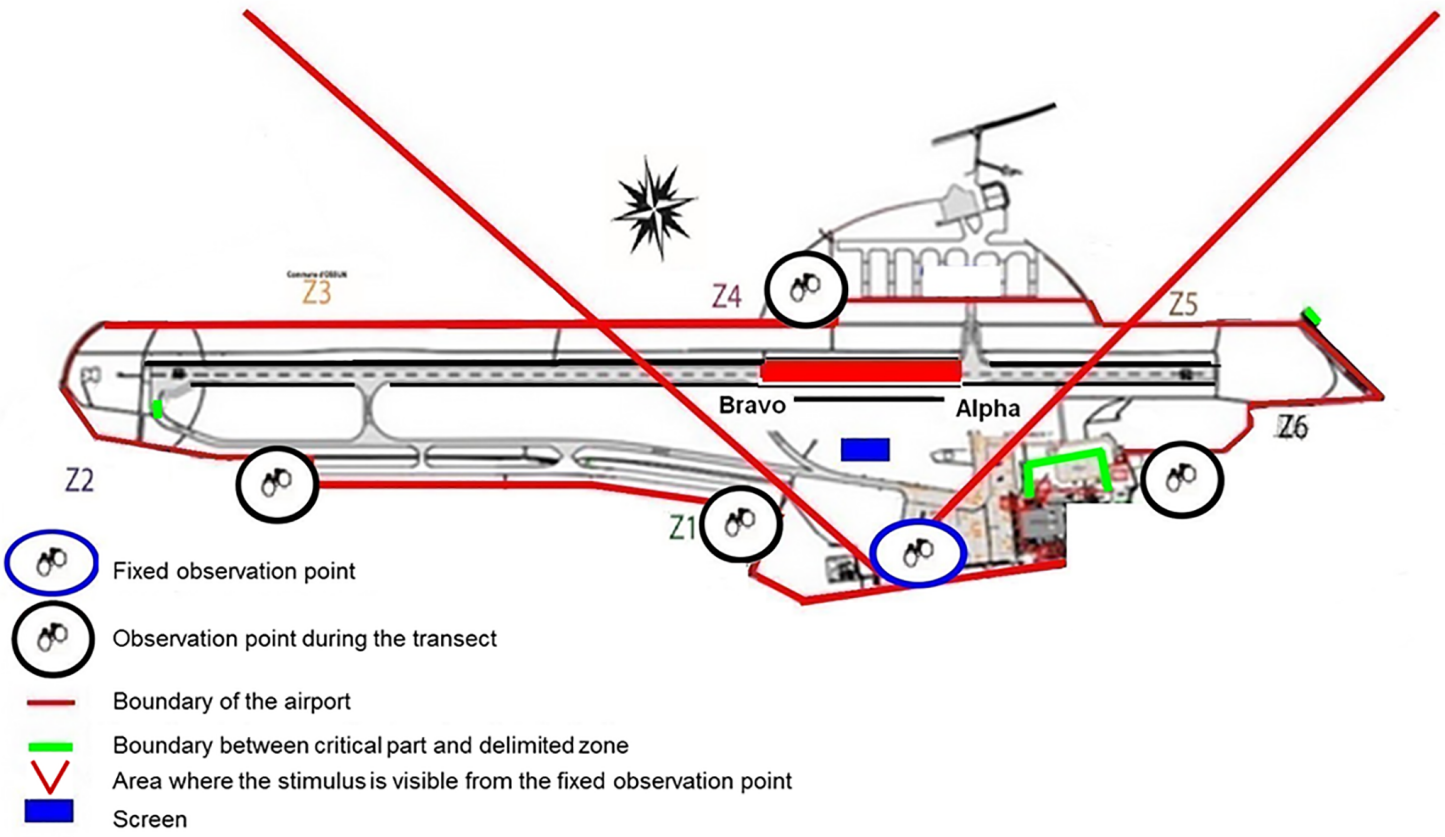
Map of the airport. a) transects used for the “entire airport” assessment. The stimuli could be seen at some places within Z1 and Z6 from a distance (> 400 m), red squares: places were observers made recordings; b) the screen area seen from the fixed observation post (within the oblique red lines), the section of the runway were the risk of collisions is the greatest lies between alpha and bravo, blue rectangle: screens.

The presence of birds in areas were the stimulus was visible and not visible were assessed using two complementary approaches using a scan sampling method [34]. A scan made every 10 min recorded the number of birds of each species was noted. All observations were carried out by the same observer (AB) (Fig 3).

1. A transect was established around the entire airport. First we listed and mapped all potential obstacles higher than a standing bird on the ground (higher than 40 cm for corvids and higher 50 cm for raptors). Visibility of the screens from the birds’ locations was assessed by the observer (AB) by verifying whether the stimulus was clearly visible from a given spot (tested by putting a camera at bird’s-eye height). Physical landmarks such as buildings, ground marks or windsocks were used to evaluate distances up to 500 m. Six observation points were determined where all birds present over a given zone (Z1, Z2 … Z6) were recorded instantaneously at each scan (Fig 3). The transect was covered by car at regular speed (10–15 km/h) once or twice a day. Covering all the transect took about 60 min. The successive observation points were reached in 10 minutes thus the interval between scans was 10min. This yielded a precise evaluation of the numbers of birds ≥ 400 m from the screens. The stimuli were then visible for flying birds and at some spots for birds on the ground.
2. Observations were made from a fixed point allowing a view over a 500 m^2^ area around the screens during two 2 h-sessions per day distributed over the daylight period (“screen area”). Within this area, screens were mostly visible but some visual obstacles (i.e. buildings, grass, hedge, mound or hollow) were present and hid the screens. Here again obstacles were measured in order to control whether a bird of a given species could see the screen or not. Ground marks and remarkable physical marks were used to evaluate distances (Fig 3).

In both cases, at each scan, we recorded all birds present whether on the ground, perched or in flight (100 meters high maximum, as assessed by the observer during a control flight in an aircraft) and specified the direction of the flight (North, South, East, West) as well as the distance to the screen either in flight or on the ground (by comparison to landmarks). Over the whole study period, we recorded 288 scans in the screen area and 239 scans along the transect yielding 3946 and 4871 bird records respectively.

In order to assess potential effects of environmental factors on the birds’ presence, temperature, humidity, wind speed and gust of wind speed were documented. All weather condition data were obtained from Tarbes—Ossun—Lourdes’ weather channel hourly daily recordings.

For the data analysis, we first run the GLM with the exact time of the day but the set of data was then too heavy and the program could not run. We then decided to consider periods of the day based on preliminary observations of the distribution of raptors which were more numerous in the morning, with a decrease at midday and a quasi-absence in the evening. Thus, three equal daytime periods were defined: morning from 7:00 to 11:00, mid-day from 12:00 to 16:00 and evening from 17:00 to 21:00. For further analysis, two periods were also determined: Period 1 from 19th to 30th August (before the stimuli were displayed) and period 2 from 30th August to 29th September (during stimuli display). Since the birds were not ringed, it was impossible to know whether the same bird was recorded several times. Therefore, comparisons between sites and time periods were numbers of birds per scan. The birds observed belonged to different groups: raptors (1967 records: mostly falcons, black kites and buzzards), corvids (1989 records: magpies, crows), passerines (3795 records), waders (825 records) (S6 Fig).

### Data and statistical analyses

The statistical analyses were performed by a researcher (CL) who was blind to the locations (zones, visibility…) and their meanings for the experiment, as well as the precise aim of the study (repelling raptors).

The captive study data were analysed in terms of number of birds reacting. Since the same birds were tested with different stimuli, we compared their effect using a Cochran test for non-independent data. We used Chi^2^ tests to estimate whether a stimulus induced more reactions than expected by chance.

The field study data were analysed using a general linear model to estimate the possible (and relative) influence of different factors on the presence and number of birds of different phylogenetic groups (raptors, corvids, passerines other than corvids and other species including waders). The model tested the impact of the stimulus on one hand and of environmental factors (weather conditions, time of day) on the other hand. The potential impact of the stimulus was estimated by a qualitative assessment: visibility. “Visibility” corresponded to times/locations when/where the stimulus could be seen by birds. Thus it included locations with visual obstacles (buildings, mounts, high grass above birds’ heads when standing on the ground) and times when the stimulus was not displayed (30^th^ August- 30^th^ September). The model therefore included stimulus visibility and environmental conditions as co-variables and the number of birds as the dependent variable. In a second step, the model was run again, adding location (observation points) in order to evaluate the potential relative weight of stimulus visibility on the spatial distribution of birds.

In line with the results of the GLM, we analysed then the influence of the stimulus by comparing the number of birds in areas of visibility versus areas of non-visibility (χ^2^tests) [36]

Data are presented as mean +/- standard error, but the statistics were performed on the raw data. Statistical significance was set at p < 0.05. All statistical analyses were run using R 3.1.1 software [37].

## Results

### Study 1: Effect of visual stimuli on captive raptors

We made and analysed 243 tests. Reactions (birds tried to escape by flying or turned their back to the screen; S2 Fig) appeared clearly within less than a second, so we could record either a reaction or no reaction. All birds reacted to one of the stimuli at least, but the number of birds reacting varied largely according to the stimulus (Cochran test for related samples, N = 27, Q = 68.60, df = 9, p < 0.00001). Only one stimulus induced significantly more reactions than expected by chance: the looming eyespots (Chi-square test, χ^2^ = 6.26, p < 0.034) (Fig 1b). All vulture species, sea eagles and black kites reacted to the looming eyespots while a few individual eagles, falcons and buzzards did not. Potential species differences could not be tested, given the low number of birds per species. However, the result was similar when only black kites (N = 5, the only species with more than two individuals) were considered. None of the birds reacted to the control grey screen and few birds overall reacted to the static stimuli. Fifty-five percent of the birds reacted to the LE stimulus by strong attempts to fly away, 33% by turning their face away, while responses to the other stimuli consisted mostly of turning to minimize visual contact (S2 and S3 Figs).

These results show that birds of prey react to 2D visual stimuli, and that a stimulus based on a contrasting “approaching” « eye shape » (black circle and white surrounds) does indeed trigger strong reactions by these birds. The hypothesis that contrast and movement are appropriate for capturing attention but also inducing avoidance in birds was confirmed for birds of prey.

### Study 2: Application to a real life setting

The GLM analysis revealed that three factors influenced strongly the presence of birds, with stimulus visibility being the most influential (raptors: χ^2^ = 255.77, dl = 3, p≤2.2 10^−16^; corvids: χ^2^ = 67.94, dl = 3, p≤1.17 10^−14^; passerines χ^2^ = 141.81, dl = 3, p≤2.0 10^−16^; other species: χ^2^ = 98.59, dl = 3, p = p≤2.2 10^−16^) and to a lesser extent the time of day (raptors: χ^2^ = 12.63, dl = 2, p = 0.001, corvids χ^2^ = 9.04, dl = 2, p = 0.01) and gust of wind speed (χ^2^ = 6.68 dl = 1, p = 0.0001 for other species). Adding location (observation point) revealed a clear interaction between visibility and location (raptors: χ^2^ = 67.11, dl = 15, p = 0.000000011, passerines X^2^ = 29.52, dl = 15, p = 0.013 and other species χ^2^ = 27.99, dl = 15, p = 0.022, a trend for corvids: χ^2^ = 23.37.5, dl = 15, p = 0.077) and some influence of the time of day (raptors: χ^2^ = 19.13, dl = 2, p = 0.00007 and corvids: χ^2^ = 12.89, dl = 2, p = 0.0016). Thus stimulus visibility influenced strongly the number of birds at the different observation points over the airport: birds were more numerous at places were the stimulus was not visible.

All birds were sensitive to stimulus visibility but the environmental factors tested had almost no influence on the birds’ presence. Therefore, we looked in more detail at the effects of stimulus display by comparing data collected over the first two weeks (screen off) to those collected the last five weeks (screen on).

**Fig 4:**
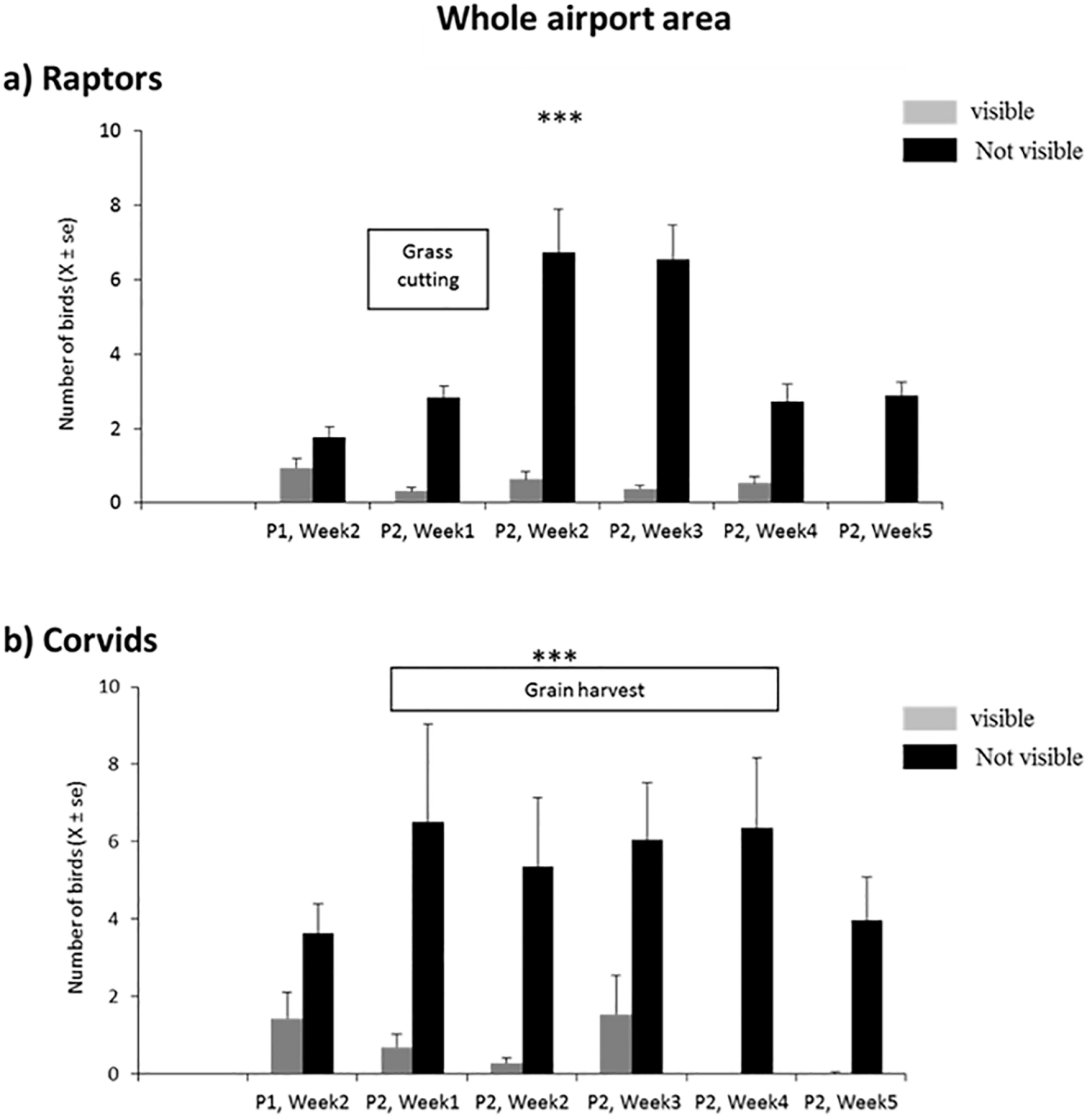
Number of birds along the transect. Numbers of birds (a: raptors, b: corvids) observed along the airport transects in relation to visual access to the stimulus (mean per scan). Chi-square test; *** p < 0.001 (n = 239 scans for the whole study period).

When considering the overall population on the airport, the total number of birds per scan increased for all bird families except waders (χ^2^, p>0.05) during the second period of observation (Chi-square test performed on real number of scans, p < 0.001 in all cases). This increase corresponded (and might have been due) to periods of grass cutting and grain harvest that attracted raptors (to feed on small mammals, invertebrates) and corvids (feeding on grain) (pers. Obs.). However, this increase of raptors and corvids was only observed in the areas where the stimuli could not be seen because of visual obstacles (Fig 4).

This relative distribution was constant over 5 weeks (χ^2^ test, Fig 4) suggesting that the birds had not become habituated over this whole period despite the continuous display of the stimulus. At the end of the experiment, corvids were even absent from areas where the stimulus was visible (i.e. had been seen by them), as though there were a sensitisation and not the usual habituation process observed, often rapidly, with recurrent presentations of deterrents.

**Fig 5:**
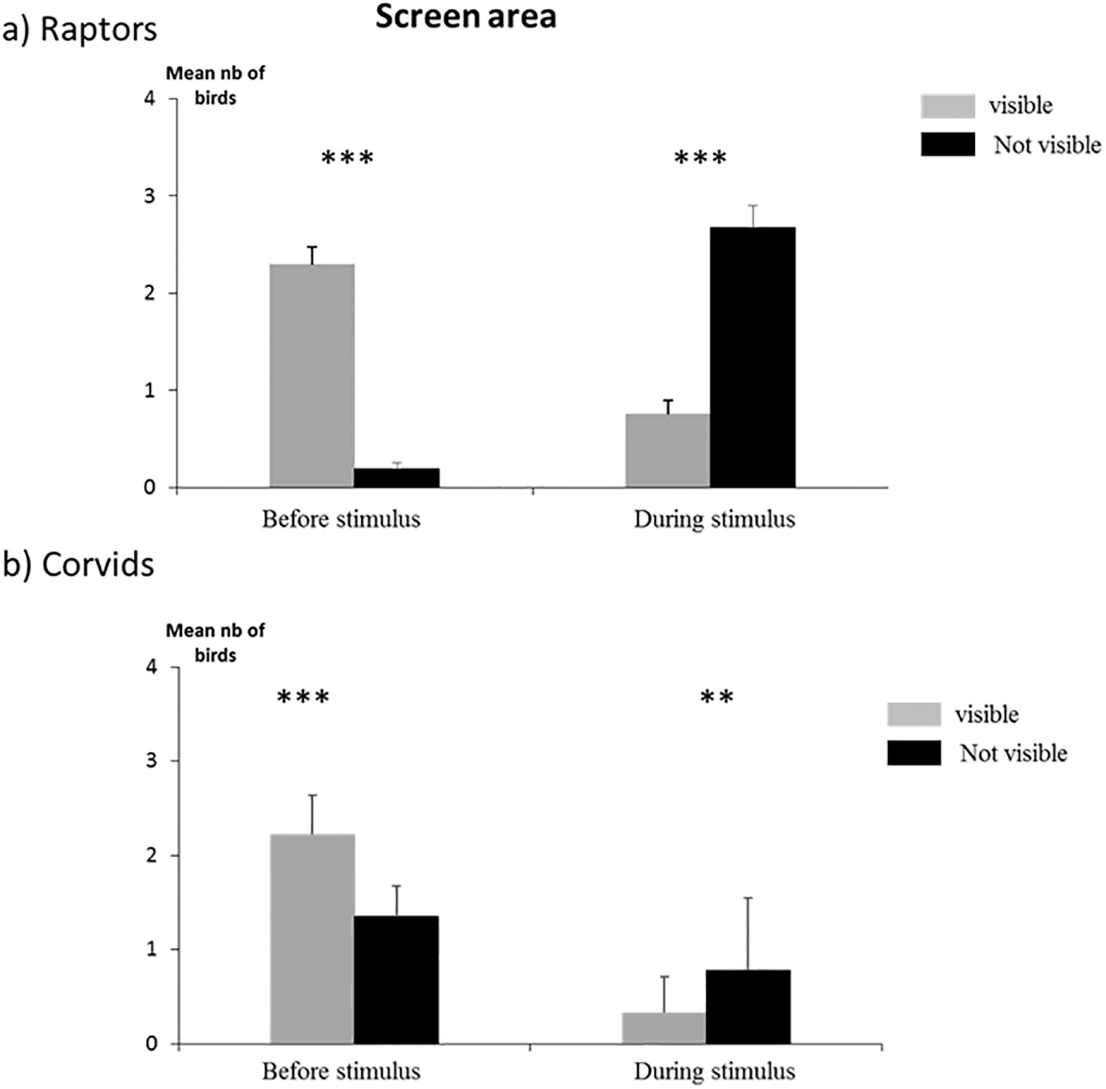
Numbers of birds in the screen area. (a: raptors, b: corvids) Numbers of birds in the screen area during the last week (before) pre-stimulus and the first week (during) the stimulus was presented for zones with or without access to the stimuli (mean per scan). Chi-square tests, *** p < 0.001 (n = 121 scans for the 2 weeks).

More precisely we observed a clear redistribution of birds in the area surrounding the screens between the last pre-stimulation and the first post-stimulation weeks; whereas most raptors observed were in the open zones in front of the screens before the stimulus was presented, only 0.8 raptors per scan were observed in these same zones when the stimulus was displayed, that is 2.8 times fewer. On the contrary their numbers increased in the visually obstructed zone (2.7 birds per scan that is 3 times more) (Fig 5). Over the whole period of display, they were 3.6 times more numerous in the obstructed than in the open zones. Similar results were obtained for the corvids: we observed 6.9 times fewer birds in the visibility zones when the stimulus was presented. Birds were more numerous (2.4 times) in the obstructed zones than in visibility zones after only one week of stimulus display (Fig 5). No such change was observed for other of bird families, such as non-corvid passerines (χ^2^, p>0.05 in all cases).

**Fig 6:**
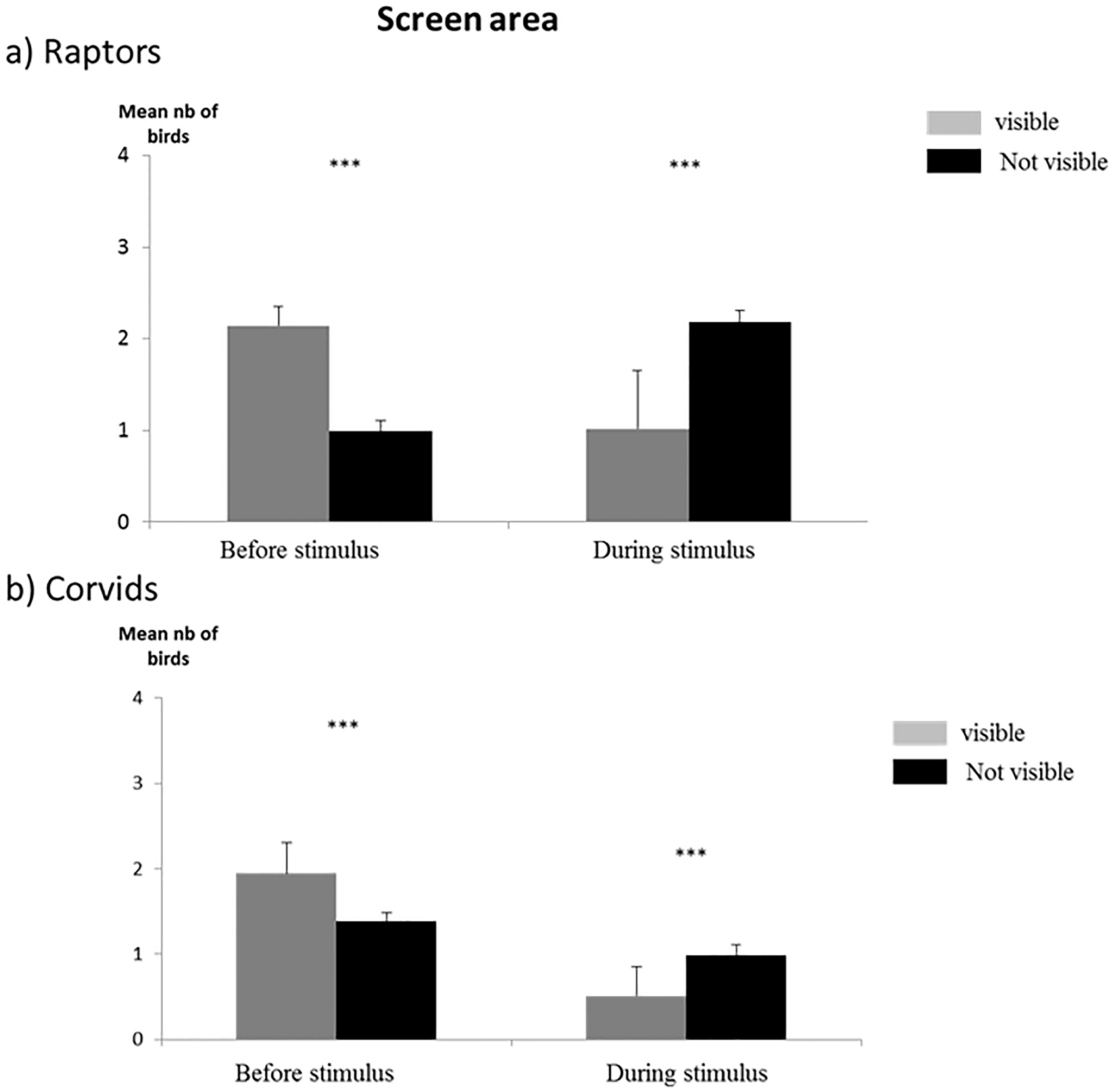
Distribution of birds. Distribution of birds over the whole study period: a) raptors and b) corvids over the screen area before and during display of the stimulus and according to visibility (mean number per scan). Chi-square test, *** p < 0.001 (n = 288 scans for the whole period).

Overall, the results for the screen area confirmed over the whole study period the data obtained at the airport scale despite the agricultural activity that increased in all areas during the period the stimulus was displayed (Fig 6). The results also show that the numbers of corvids overall diminished in the whole screen area while their numbers increased during that same period on the entire airport surface.

## Discussion

This experimental study performed under controlled conditions on captive raptors confirmed the hypothesis that a “superstimulus” based on eye-shape characteristics combined with a looming effect may induce strong avoidance reactions. The results of our statistical model obtained in the field case study performed in an airport where raptors are abundant converge to show that raptors, but also corvids, were sensitive to this « looming eyes » stimulus and avoided areas where they were visually confronted with it. The results based on more than 8800 records of birds, therefore strongly suggest that the continuous display of large looming eyespots repelled birds of prey and corvids from undesirable areas. The signal obviously acted as aversive, discouraging birds from occupying locations where they could see it. Our results for this airport suggest a high potential for application. Although they are based on only one case study (but an extensive data set), the differences in the birds’ spatial distribution according to stimulus visibility are significant and did not show habituation the stimulus over the 5 weeks of experiment. Further development will have of course to involve further sites. Precise observations for the area immediately surrounding the screen show that both raptors and corvids concentrated on the smaller, less attractive (less grass) zones where the stimulus could not be seen. The responses of tethered captive raptors to this stimulus were either attempts to fly away or turning their back to it, so not to see it. This was not the case for other bird species such as passerines or waders, and this finding confirms earlier studies showing a brief or no frightening effect of eyespots on different passerine species [10, 35] even when this stimulus is associated with a looming movement (Belin et al. in prep). This may be due to differences in the visual system between species. Raptors have a very narrow binocular visual field with no stereopsis: this means that signals that generate redundancy by stimulating both eyes may be detected better [15]. In this sense, the two separate looming circles which constitute the « eyes stimulus » may have been perceived by both eyes. Also, the stimulus was in agreement with Martin’s [15] suggestion that a deterrent visual image has to be moving and large, especially in relation to raptors’ visual system, specialised for movement detection. On the other hand, jackdaws at least have been repelled from nests through photographs of eyes [38]. One cannot therefore totally exclude that the stimulus has really been perceived as two big approaching eyes, but why this might scare raptors and corvids as much as other bird species is worth of further experimental investigation. Few deterrent systems differentiate between types of birds and hence this LE stimulus may be especially useful where raptors have to be deterred from other facilities for example [12]. The design of the eyespots was based on Stevens et al.’s [19] «conspicuous signal hypothesis» suggesting that contrast and size are the most important features, which we maximized by using a black central circle within a white surround in the field study. They also showed that square contours are as efficient as round contours which made the use of a «classical» LCD screen easier.

Our field data showed that the birds permanently avoided the areas where the stimulus was visible. This is a very important conservation issue as it means that birds are just excluded from undesired areas without being harmed and seemingly without excessive stress. The fact that a few birds remained in the zones of visibility (about 1 every 2 scans) could indicate that some birds are resistant to the stimulation either because they are particularly fearless, cannot see well or have developed other avoidance strategies. Thus, a few birds kept perching on nearby poles but turned away from the stimulus (and hence also to the runway), constructing their own visual obstacle. This recalls strategies shown by captive raptors. Both « natural » eyespots and looming images do not seem to induce habituation [39, 23] therefore combining them may have been the key for the long-term effects observed with even a possible sensitisation for corvids. Because the procedure appeared stressful for the captive birds, we did not repeat the experiment with the same birds, which would be the only way to test potential sensitisation, i.e. increase of response instead of the decrease observed with habituation. This deserves further consideration. The captive raptors reacted to the visual stimuli within a time span (less than a second) that is in accordance with the « time to collision » response of the looming neurons [25] (800 to 1400 ms). The permanent display of a looming stimulus may have repeatedly triggered these looming neurons abnormally often, inducing an aversive response. Here again, only further neurophysiological studies could test this hypothesis.

Despite limitations due to a single case field test, this study (according to our knowledge [12]) was the first to demonstrate, on the basis of both captive and field studies and extensive data collection, the efficiency of a visual non-invasive stimulus to repel birds of prey and corvids from danger areas on an airport. The visual « superstimulus » appeared much more resistant than any other system to habituation despite continuous presentation over 5 weeks and induced an aversion for the zones where the signal was visible. Indeed, devices such as human silhouettes [40] or balloon with eyespots [41] used to repel birds induced rapid habituation respectively 4 days for corvids and 3 weeks for smaller passerines (starlings, blackbirds). Our stimulus may therefore constitute the first effective system to prevent raptors’ and corvids’ strikes on airports. This is promising for airports which attract these large birds because of local resources [42].

These findings open promising new applications for human security and bird conservation, as well as for research on avian visual perception and sensitivity to signals.

## Ethical statement

These studies comply with the French laws related to animal experimentation and the European directive 86/609/CEE and 2010/63/UE and were approved by the University of Rennes 1 local Animal Care Committee. No specific permissions were required for these experiments. (The captive birds’ husbandry and care were under the management of the private facility: none of the birds used was a research animal and the owner of the facility gave permission to conduct the study on this site).

## Supporting information

S1 Fig The nine visual stimuli presented to raptors. (TIF)

S2 Fig Example of reaction of a captive raptor (Turkey vulture *Cathartes aura*) to looming eyes stimulus. (AVI)

S3 Fig Example of reaction of a captive raptor (Turkey vulture *Cathartes aura*) to a stripe shape stimulus. (AVI)

S4 Fig Example of presence of raptors on runway before stimulus display. (JPG)

S5 Fig Location of the screens according to the runway. Red strips indicated the area to be protected.
(JPG)

S6 Fig List of bird species present at the airport. (JPG)
