# Supplementary figures and images for: Wide-eyed glare scares raptors: From laboratory evidence to applied management
Source: PLoS One. 2018 Oct 11;13(10):e0204802. doi: 10.1371/journal.pone.0204802 (PMC6181303; doi:10.1371/journal.pone.0204802)

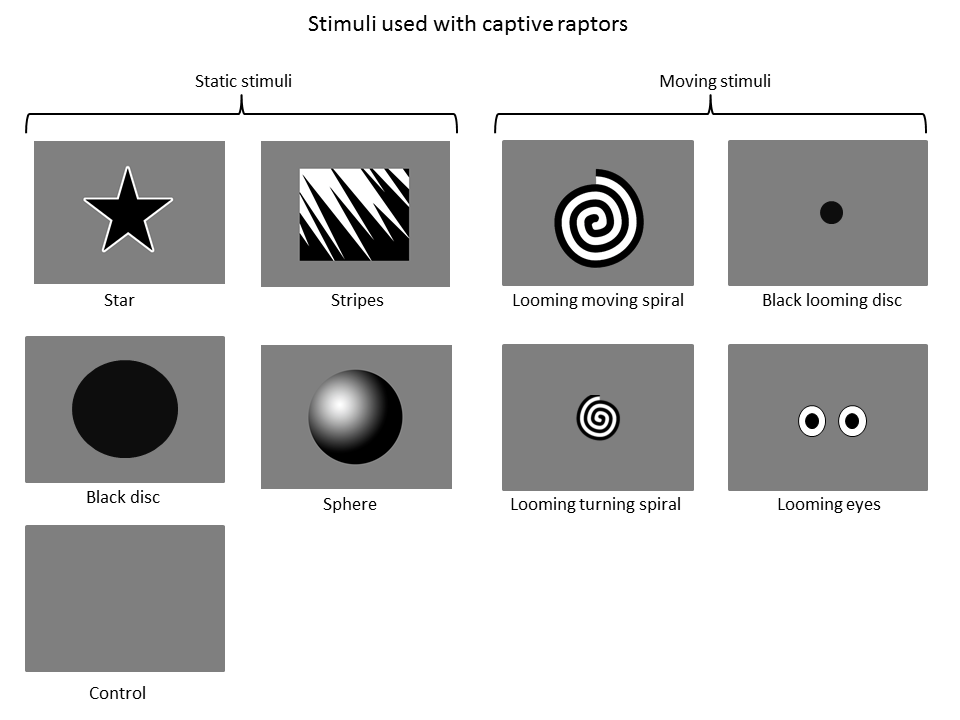

Supplement: S1 Fig — (TIF) [file pone.0204802.s001.tif]

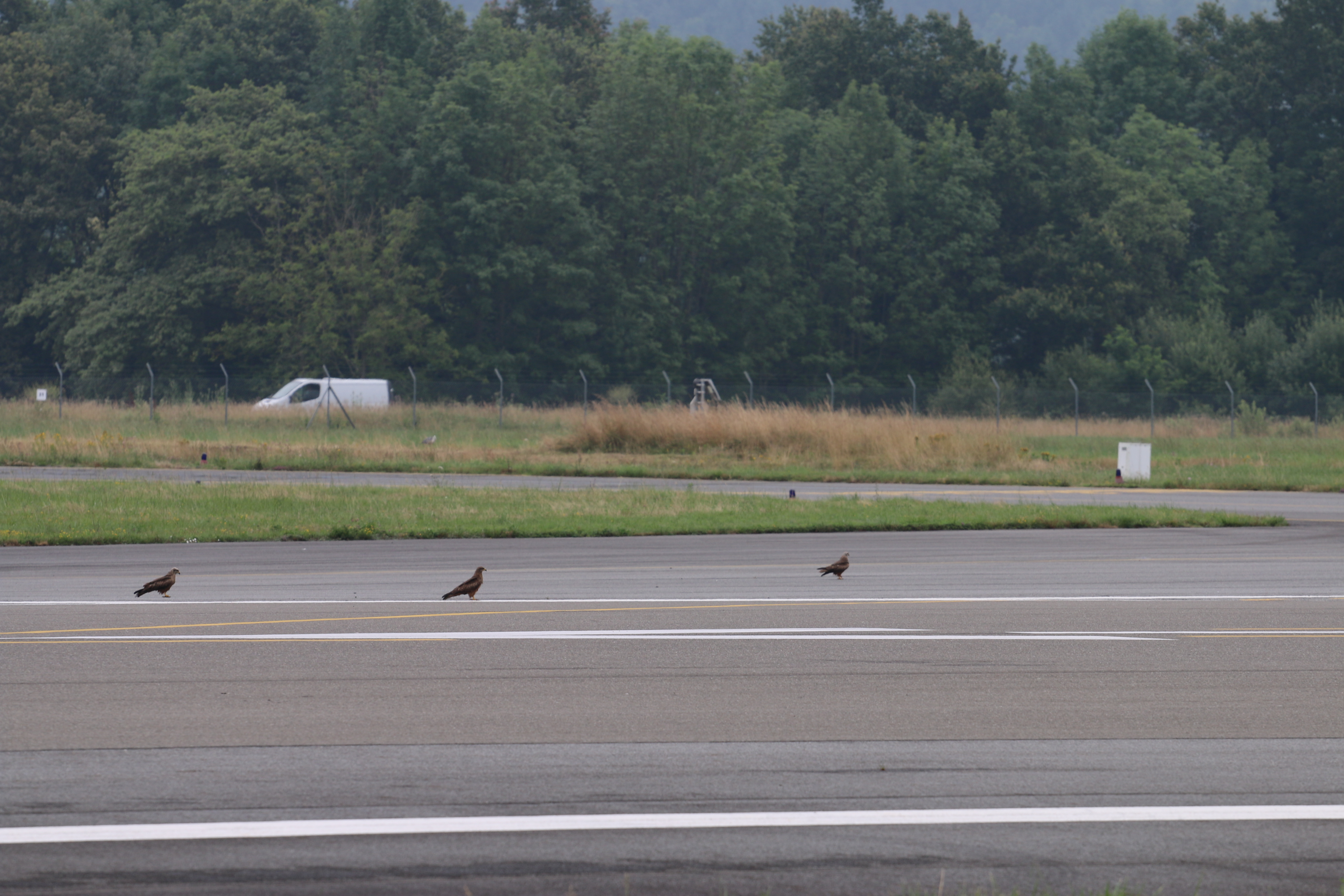

Supplement: S4 Fig — (JPG) [file pone.0204802.s004.JPG]

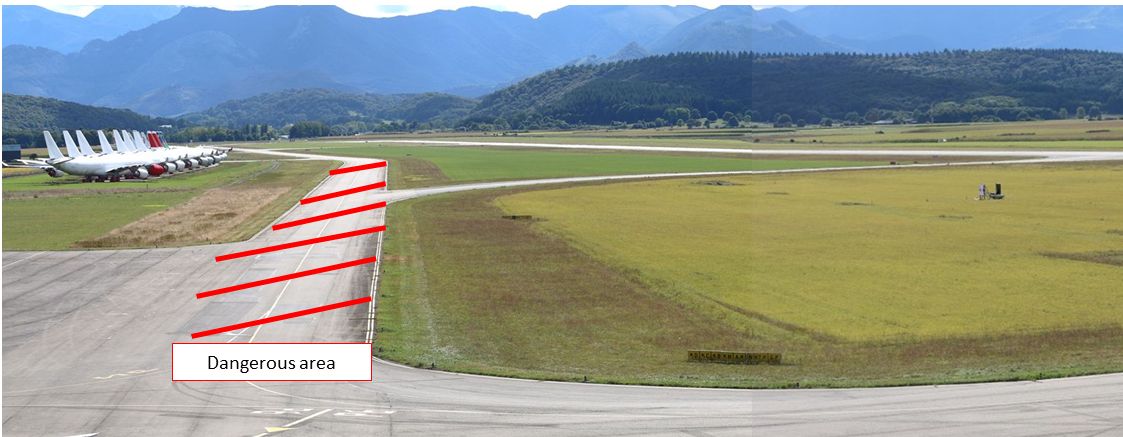

Supplement: S5 Fig — Red strips indicated the area to be protected. (JPG) [file pone.0204802.s005.jpg]

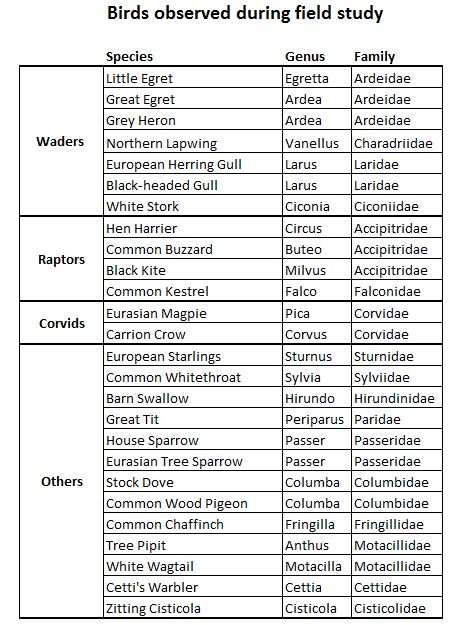

Supplement: S6 Fig — (JPG) [file pone.0204802.s006.jpg]
